# Antitumor Potential of Antiepileptic Drugs in Human Glioblastoma: Pharmacological Targets and Clinical Benefits

**DOI:** 10.3390/biomedicines11020582

**Published:** 2023-02-16

**Authors:** Manuela Stella, Giammarco Baiardi, Stefano Pasquariello, Fabio Sacco, Irene Dellacasagrande, Alessandro Corsaro, Francesca Mattioli, Federica Barbieri

**Affiliations:** 1 Pharmacology and Toxicology Unit, Department of Internal Medicine, University of Genova, 16132 Genova, Italy; 2 Clinical Pharmacology Unit, EO Ospedali Galliera, 16128 Genova, Italy

**Keywords:** brain tumor, glioblastoma, antiepileptic drug, anti-cancer therapy, cancer neuroscience

## Abstract

Glioblastoma (GBM) is characterized by fast-growing cells, genetic and phenotypic heterogeneity, and radio-chemo-therapy resistance, contributing to its dismal prognosis. Various medical comorbidities are associated with the natural history of GBM. The most disabling and greatly affecting patients’ quality of life are neurodegeneration, cognitive impairment, and GBM-related epilepsy (GRE). Hallmarks of GBM include molecular intrinsic mediators and pathways, but emerging evidence supports the key role of non-malignant cells within the tumor microenvironment in GBM aggressive behavior. In this context, hyper-excitability of neurons, mediated by glutamatergic and GABAergic imbalance, contributing to GBM growth strengthens the cancer-nervous system crosstalk. Pathogenic mechanisms, clinical features, and pharmacological management of GRE with antiepileptic drugs (AEDs) and their interactions are poorly explored, yet it is a potentially promising field of research in cancer neuroscience. The present review summarizes emerging cooperative mechanisms in oncogenesis and epileptogenesis, focusing on the neuron-to-glioma interface. The main effects and efficacy of selected AEDs used in the management of GRE are discussed in this paper, as well as their potential beneficial activity as antitumor treatment. Overall, although still many unclear processes overlapping in GBM growth and seizure onset need to be elucidated, this review focuses on the intriguing targeting of GBM-neuron mutual interactions to improve the outcome of the so challenging to treat GBM.

## 1. Introduction

Glioblastoma (GBM) is the most common primary malignant CNS tumor (48%) [1]. In the United States, the average annual age-adjusted incidence of GBM is 3.2/100,000 [1], while in Europe, it ranges from 3.3/100,000 in France [2] to 4.17/100,000 in Spain [3]. GBM occurs mainly in older adults (>65 years), and the male:female ratio is 1.6:1 [4,5], and the global incidence of GBM increases with age [6]. GBM is an astrocytic tumor characterized by high cellular heterogeneity, invasiveness, and microvascular proliferation. All these characteristics concur in patients’ short median survival and frequent recurrence, also favored by an immunosuppressive tumor-induced microenvironment fostering the self-renewal of highly infiltrative and drug-resistant GBM stem cells (GSCs) in the peritumoral region [7].

In 2016, the World Health Organization (WHO) newly classified CNS tumors by increasing the importance of their molecular profile in addition to histopathologic features; GBM is defined as a WHO grade IV glioma [8]. The 2021 WHO classification further expanded the relevance of molecular diagnostics, adding isocitrate dehydrogenase (IDH) mutational status and 1p/19q co-deletion to better define glioma subtypes; currently, a diffuse astrocytic glioma with microvascular proliferation, necrosis, and/or specific gene alterations (i.e., TERT mutation, epidermal growth factor receptor (EGFR) amplification, and chromosome rearrangement, IDH*^wt^* without 1p/19q co-deletion) is diagnosed as GBM WHO grade 4 [9].

Despite continuous advances in our understanding of GBM pathogenesis, from self-renewal of GSCs [10] to pro-tumorigenic microenvironment [11] or epigenetic phenomena [12] driving tumor progression, the prognosis of affected patients remains poor. Since 2005, standard of care (SOC) therapy consists of surgical resection, concurrent radiotherapy (RT, total 60 Gy) with the alkylating agent temozolomide (TMZ, 75 mg/m^2^/day for 6 wk), and further adjuvant TMZ (150–200 mg/m^2^/day for 5 days for six 28-day cycles) [13], ensuring an overall survival (OS) of approximately 15 months in newly diagnosed GBM [14]. Tumor-treating fields can also be given concurrently with adjuvant TMZ [15].

Methylation of the O^6^-methylguanine-DNA methyltransferase (MGMT) promoter is the strongest predictor for benefit from TMZ; thus, this drug could be withheld in selected patients with unmethylated MGMT tumors [13]. Neither the extension of TMZ treatment nor the addition of bevacizumab, a humanized anti-VEGF monoclonal antibody, yielded further survival benefits for newly diagnosed GBM [16,17].

Recurrence is nearly inevitable after a median interval of ~7 months [18], and agreement on SOC is lacking [19]. Since the efficacy of current treatment for the recurrent disease remains poor, the National Comprehensive Cancer Network (NCCN) recommends enrollment in clinical trials as the preferred therapeutic approach [20]. Surgery may have a role for symptomatic lesions, while other treatment options include re-irradiation, TMZ rechallenge, PCV (procarbazine, CCNU [lomustine], vincristine), bevacizumab, nitrosourea, or target therapies [20]. TMZ rechallenge may be a reasonable option for MGMT methylated GBMs [21,22], while bevacizumab, often effective in reducing clinical symptoms related to peritumoral edema, failed to demonstrate survival benefit [23]. Despite progress made in developing targeted therapies [24], none has been shown to prolong survival in randomized clinical trials [14,19]. Immunotherapies (vaccination, immune checkpoint blockade, oncolytic viral therapies, chimeric antigen receptor T-cell therapies) are a growing perspective, despite the fact that their potential efficacy needs to be demonstrated [25].

## 2. Glioblastoma-Related Epilepsy (GRE)

Glioma patients’ quality of life largely depends on the neurological decline due to the tumor itself or treatment-related toxicity and epilepsy (for definition, see [26]), which negatively affects neurocognitive functions and outcome [27].

In GBM patients, the diagnosis of epilepsy is usually made after one seizure episode. An inverse relationship between the degree of malignancy and the frequency of seizures has been reported: patients with low-grade glioma are affected by seizures in 65–95% of cases, while they occur in 30–50% of GBM patients [28]. GBM often originates in less epileptogenic areas, and patients may not live long enough to develop epilepsy, thus explaining the relative lower incidence of epilepsy in these tumors [29,30]. Multiple factors concur to GRE onset, such as increased intracranial pressure and edema, alterations of the peritumoral cortex, inflammation, and vascular insufficiency, likely triggering excitotoxicity and hyperexcitability of peritumoral neurons [31]. Underlying molecular mechanisms involve disruption of inhibitory/excitatory transmission balance mainly due to alterations of GABAergic and glutamatergic regulation [31], whose functions connecting epileptogenicity and GBM progression are detailed in the following paragraphs. The involvement of glioma-derived thrombospondin, a regulator of synaptogenesis, in excitatory synapse formation within the peritumoral cortex of a glioma-cell-implanted rat model has been described [32]. Furthermore, the association between connexin 43, a multifunction protein that forms gap junction channels, and GRE has been suggested and discussed [33,34].

Based on the relevance of alterations in GBM surrounding tissue in GRE, the pathophysiology of epilepsy may have roots in the tumor microenvironment; indeed, in the peritumoral area from a rat model of glioma, the density of GABAergic neurons was significantly decreased, and transcriptomic analysis revealed that 5 of 19 genes differentially expressed were associated with epilepsy and neurodevelopmental disorders [35].

The presence of GRE is considered one of the major risk factors for long-term disability since antiepileptic drugs often have heavy side effects and interactions with chemotherapy and supportive therapy; therefore, further investigation on neurons within the GBM microenvironment will identify targetable mechanisms in a translational perspective.

## 3. Antiepileptic Drugs in GRE: Clinical Management

Seizure control in GBM patients can be hardly accomplished by surgery alone; however, the extent of resection may determine better seizure outcome [36], while a beneficial effect of radio-chemo-therapy has not yet been defined: it seems effective for low-grade glioma [37] but not in GBM elderly patients [38]. Therefore, the best treatment strategy of GRE with AEDs can guarantee epilepsy control with a great impact on the patient’s quality of life. Management of GRE with a specific AED is challenging and controversial. Considering the risk of AED adverse events, physical disability, and neurocognitive impairments related to the tumor site, a multidisciplinary approach is needed.

To date, no universal guidelines are available for GRE therapy, and the antiepileptic efficacy of drugs in non-tumor epilepsy is not directly translatable to GBM patients due to drug–drug interactions and neurological conditions. In GRE, no superior efficacy of one AED over another has been demonstrated [39,40], and we are waiting for results from ongoing trials (NCT03048084, NCT03636958, NCT04497142). Current recommendations on AEDs use in newly diagnosed GBM suggest not treating patients who have never experienced a seizure episode, while it should be prescribed for patients who had at least one comitial episode to prevent the high risk of recurrent seizures [40,41]. Nevertheless, this issue is still discussed [42], and currently, refractory seizures may be treated with the combination of AEDs with a different mechanism of action [39].

The non-enzyme inducing AEDs, levetiracetam (LEV), lamotrigine (LMG), topiramate (TPM), lacosamide (LCM), pregabalin (PRG), and valproic acid (VPA) are preferred as monotherapy, as they have fewer adverse effects and interactions with other concomitant therapies in cancer patients. VPA acts as an inhibitor of glucuronidation; TPM is a weak inducer of CYP3A4 and might also inhibit CYP2C19. Perampanel (PER) and brivaracetam (BRV) are potentially broad-spectrum antiepileptic drugs that may be useful in add-on and do not appear to act as potent inducers of cytochrome P450 isoenzymes [43]. However, VPA, TPM, and PER can induce enzyme inhibition, increasing the toxicity of antitumor substrates. [44,45].

The use of enzyme-inducing AEDs (phenytoin, PHT, phenobarbital, and carbamazepine) is generally discouraged as they are potent inducers of several CytP450 isoenzymes, leading to clinically relevant alteration of antitumor agent pharmacokinetics (e.g., lomustine, vincristine), possibly increasing their side effects and reducing efficacy [40]. When a drug interaction is suspected, AEDs serum concentrations should be monitored [46]. However, taking into account that the metabolism of TMZ is not affected by old AEDs, the main reason for their limited use is the higher safety, tolerability, and effectiveness of newer AEDs [47].

A systematic review demonstrates that monotherapy with LEV, PHT, and PRG had higher efficacy in GRE, with LEV showing a lower failure rate [47], although variability among studies and patient populations prevents a definite statement. LEV shows a more favorable efficacy profile compared to VPA, with similar toxicity [47,48]. 

Drug resistance to first-line monotherapy in patients with GRE occurs in approximately 30% of patients and requires an add-on. However, no evidence of the superiority of a drug over another in resistant patients has been reported [40]. Among add-on treatments, PER efficacy and safety in GRE have been described [49,50], as well as for BRV [51], an analog of LEV, currently under evaluation (NCT05029960), although larger prospective studies are lacking.

Given the emerging interest in cannabinoids (i.e., cannabidiol, CBD) as promising antiepileptic agents in patients with refractory seizures [52], CBD-enriched products might help control seizures also in glioma patients. CBD showed good safety and tolerability [53], while clinical efficacy in GRE needs to be tested. 

Therapeutic criteria for GRE are still based on studies on general epilepsy; thus, a satisfactory GRE therapy is still challenging. In this scenario, further insights into specific pathophysiological mechanisms underpinning GRE and drug resistance might improve AED choice and patient outcome.

## 4. Molecules at the Crossroads of GBM Development and Epilepsy

Many mechanisms cooperate and overlap in tumorigenesis and epileptogenesis, which mainly impact GRE pathophysiology. Indeed, GRE shows a definite clinical profile but implies several causes related to both the patient’s (age, genetic factors, therapy, physical status) and tumor (subtype, location, infiltration in the brain parenchyma, edema, microenvironment, neoangiogenesis, inflammation, infiltrating microglia) features.

At the molecular level, the epileptogenic-related process includes IDH mutation status, mTOR signaling pathway, and neurotransmitter (GABA, glutamate) dysregulation—all shared relevant mechanisms in GBM development.

### 4.1. Intrinsic Molecular Features of GBM Progression and Their Role in GRE

IDH enzymes participate in the metabolic process and cellular homeostasis catalyzing the oxidative decarboxylation of isocitrate. Mutations in IDHs are frequent in a variety of human malignancies, including gliomas, which correlate with seizure risk [54]. Preoperative epilepsy is more frequent in IDH*^wt^* low-grade glioma patients than in IDH*^wt^* GBM [55].

IDH mutation likely leads to the formation of D-2HG (D-2-hydroxyglutarate), an oncometabolite structurally similar to glutamate, able to enhance the activity of tumor-adjacent cortical neurons interacting with NMDA receptors and, therefore, boosting seizures [56]. In IDH*^mut^* tumors, high D-2HG concentration leads to metabolic disruptions in surrounding cortical neurons that consequently promote seizures, increasing preoperative seizure risk. Further evidence suggests that D-2HG supports a metabolic change (hypermetabolic phenotype) in GBM surrounding neurons, characterized by lactic dehydrogenase-A increase, defects in the tricarboxylic acid cycle [56,57], as well as upregulation of mTOR (mechanistic target of rapamycin) activity [56,58], already known as pro-epileptogenic stimuli. Indeed, mTOR signaling is responsible for cell growth and survival, and alterations are related to multiple brain disorders, including neoplasms and epilepsy [59]. Interestingly, in GBM, the PI3K/AKT/mTOR pathway is almost uniformly activated due to activating mutations in PIK3CA or PIK3R1 or loss of phosphatase and TENsin homolog (PTEN) [60,61]. Dysfunction of the mTOR pathway also modifies GABA and glutamate signaling, two main actors in epileptogenesis [62].

Furthermore, mTOR, the most intrinsic properties of GBM, depicting its specific molecular profile, directly influences the development of epilepsy. Mutations in tumor suppressor genes, such as tumor protein 53 (TP53), PTEN, and neurofibromatosis type 1 (NF1), whose downstream altered pathways drive GBM development, are also closely related to peritumoral hyperexcitability, as observed in epileptogenic GBMs developed in a PTEN, NF1, and p53 CRISPR-based animal model [63,64,65]. Frequent TP53 mutations in GBM lead to upregulation of the protein, which in turn triggers MET and EGFR activity, enhancing a tumor invasive behavior and matching with TP53 overexpression associated with preoperative seizures [66]. Similarly, PTEN mutations causing dysregulation of the mTOR pathway result in epileptic disorders [67,68].

Pro-epileptogenic effects of NF1 mutations are evident in patients bearing this multisystem disorder, commonly leading to brain tumor predisposition and neurological manifestations, in which seizures occur in 4–7% of patients [69]. In GBM, neurofibromin, the NF1 gene product, negatively regulates mTOR signaling through downregulation of the Ras/MAPK pathway, hyperactivation of mTOR, and increased cell proliferation, favoring tumor progression [70]. Notably, alterations of the RTK/RAS/PI3K pathway, found in 90% of GBM, are also driven by mutations in the PI3K catalytic subunit PIK3CA [60], as described in patient-derived xenograft mice models harboring mutant PIK3CA, in which gliomagenesis and neuronal hyperactivity showed a reciprocal influence, likely through a secreted factor (e.g., glypican 3, GPC3), selectively expressed in these tumors [71]. A complementary activity of secreted molecules involved in neuronal activity, such as neuroligin-3 or glutamate, has been associated with GBM growth [72].

Overall, the intrinsic molecular features of GBM share most of their activities with epilepsy, affecting both tumor and neuronal behavior.

### 4.2. Neurotransmitter Synaptic Inputs to GBM Progression and GRE

**Figure 1:**
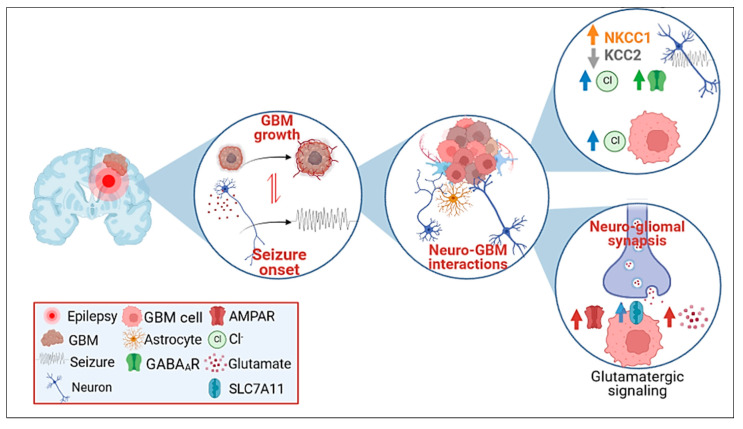
Neuro-glioma interplay: role in glioblastoma (GBM) progression and in GBM-related epilepsy. (Details of the molecular processes are described in the text). The schematic diagram depicts the interactions between neurons and GBM cells, leading to seizure onset and GBM growth, focusing on the role of neurotransmitters (GABA and glutamate) imbalance. In neurons of epileptogenic tissue, upregulation of NKCC1 (Na–K–2Cl cotransporter (1), and the concomitant low expression of KCC2 (K–2Cl symporter (2), which normally control the low intracellular Cl^−^ concentration required for GABA_A_R-mediated inhibition, is significantly decreased. Consequently, intracellular Cl^−^ concentration increases and triggers a depolarizing GABAergic excitatory response firing seizure onset. In parallel, GBM shows perturbation of Cl^−^ homeostasis accumulating high concentrations of Cl^−^. The neuro-gliomal synapses lead to interactions mediated by glutamate receptors (AMPAR) and overlapping mechanisms involved in both oncogenesis and epileptogenesis. GBM cell express calcium-permeable AMPAR, which are stimulated by glutamate in an autocrine (GBM cells release a high amount of glutamate) and paracrine (neuronal) manner promoting GBM cell proliferation and invasion. This mechanism is mainly mediated by high upregulation of the cystine/glutamate antiporter SLC7A11 (Solute Carrier Family 7 Member 11) on the GBM cell synaptic membrane, which associates with seizure onset.

Cancer cells may co-opt neuronal signaling pathway, and reciprocally, neuronal activity may drive GBM malignant behavior by either autocrine or paracrine (non-synaptic) mechanisms. GRE is often linked to unbalanced inhibitory and excitatory synaptic signals leading to excessive excitability in GBM surrounding tissues [73]. Therefore, the involvement of GABAergic and glutamatergic systems, which maintain a finely-tuned excitation-inhibition balance in normal tissue, deserves particular interest in oncogenesis and epileptogenesis (Figure 1).

### 4.3. GABAergic Signaling

The inhibitory GABA transmission is mediated by ligand-gated chloride-permeable channel GABA_A_ receptors (GABA_A_Rs), and the response type (depolarization vs. hyperpolarization) is mainly driven by intracellular Cl^−^ concentrations in post-synaptic neurons, regulated by a neuronal-specific solute carrier (SLC) family protein 12 (SLC12A), the member of the K^+^-Cl^−^ cotransporter 2, KCC2 [74] (Figure 1).

Surrounding neocortical cells in epilepsy-associated lesions show downregulation of GABA_A_Rs and reduced synapses, likely leading to unbalance between excitatory and inhibitory transmission, which, in turn, may contribute to the pathogenesis of focal seizures [75]. As in other human focal epilepsies, pathologic changes in Cl^−^ homeostasis can switch GABAergic signaling from hyperpolarizing to depolarizing. Excitatory effects of GABA in the human peritumoral neocortex contribute to epileptogenic glioma [76].

Epileptogenicity of GABAergic neurotransmission in GBM seems to be linked to dysregulation of two cation-chloride cotransporters: (i) downregulation of KCC2, which is usually expressed at high levels in cerebral tissue and extrude Cl^−^; and (ii) upregulation of Na–K–2Cl cotransporter 1 (NKCC1), which is normally expressed at low levels actively uptakes Cl^−^ into cells [76,77]. This process leads to an increase in neuronal intracellular Cl^−^ concentration, which, contrarily to what is observed in a normal brain (hyperpolarization), shifts towards a depolarizing GABAergic excitatory response [62,78], contributing to GRE (Figure 1). KCC2 is downregulated in the peritumoral region while NKCC1 is highly expressed [79], and gliomas show perturbation of Cl^−^ homeostasis since GBM cells accumulate Cl^−^ through the activity of NKCC1 transporter, supporting their proliferative and migratory ability [80] (Figure 1).

### 4.4. Glutamatergic Signaling

**Figure 2:**
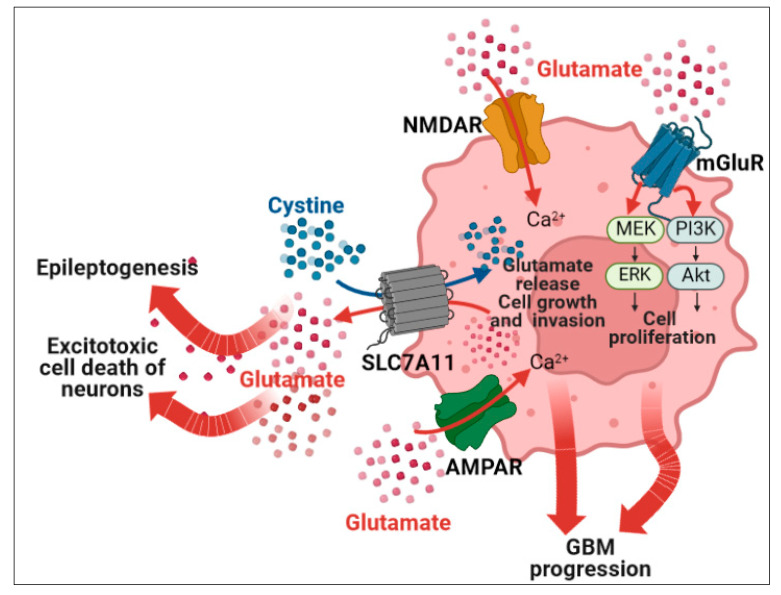
Main glutamatergic signaling in glioblastoma cells and peritumoral neurons leads to tumor progression and epileptic onset. SLC7A11 (cystine/glutamate antiporter solute carrier family 7 member 11) is often upregulated in glioblastoma (GBM) and associated with seizures. GBM cells release glutamate via SLC7A11 in exchange for cysteine (precursor glutathione which mitigates oxidative stress and sustains GBM cell survival) uptake. The abundant release of glutamate concurs to enhance the excitotoxicity of GBM surrounding neurons and contributes to tumor growth in a paracrine manner. The upregulated glutamatergic activity in neurons stimulates glioma growth and signaling via ionotropic glutamate AMPA and NMDA receptors (AMPAR and NMDAR), which are also involved in seizure onset. GluR2 subunit-lacking AMPARs are highly Ca^2+^ permeable leading to increased Ca^2+^ influx in GBM cells that (a) enhances glutamate release to surrounding cells, (b) triggers growth- and invasion-related pathways in GBM cells, (c) impacts neuro-gliomal glutamatergic synapses. NMDARs are highly expressed in synapses but also in glia and GBM cells. NMDARs concur to increase intracellular Ca^2+^ levels via activation of mGluRs glutamatergic transmission in both neurons and glia, and glioma. Metabotropic glutamate receptors (mGluRs) are G-protein-coupled receptors that affect ion exchange when activated by glutamate and regulate major pro-survival and proliferative signaling pathways such as MEK/ERK (mitogen-activated protein kinase kinase 1, MEK; extracellular signal-regulated kinase ERK), and PI3K/Akt (phosphatidylinositol-3 kinase, PI3K; serine/threonine kinase, Akt).

In addition to GABA signal disruption, glutamatergic mechanisms, influencing intracellular Cl^−^ concentration, and imbalance of the excitatory neurotransmitter glutamate in neurons, contribute to cell excitability, the pathophysiology of seizures, and epileptogenicity in the tumor-surrounding tissue (Figure 1). Ionotropic glutamate AMPA and NMDA receptors are predominantly involved in seizure onset [81]. Interestingly, in the glioma microenvironment, in addition to neuron-released glutamate, GBM cells, contrarily to normal astrocytes which sequester the neurotransmitter, release glutamate, likely exerting a pro-epileptic activity in the peritumoral brain (Figure 2). Insights into glutamatergic interactions in glioma are increasing: GBM cells express high levels of SLC7A11solute carrier family 7 member 11or xCT, a subunit of xc-cystine-glutamate transporter system, which acts as a cystine/glutamate antiporter across the plasma membrane. Increased exchange of extracellular cystine and intracellular glutamate induces glutathione synthesis, concurring either endogenous antioxidant activity sustaining GBM cell survival or glutamate release (Figure 2). SLC7A11 upregulation correlates with tumor invasion and outcome in GBM patients as well as with the onset of seizures, as described in animal models of human GBM xenografts implanted into the mouse brain [82,83], although the seizures in these experimental animals could not be completely representative of rhythmic and periodic patterns of seizure in humans. However, Buckingham et al. [83] describe a novel GBM-related pathophysiological mechanism of seizure onset, in which glutamate released by the GBM cells through SLC7A11 causes hyper-excitability of surrounding neurons. Surgical removal of GBM mass might improve GRE by reducing glutamate-releasing GBM cells. 

GBMs also show the downregulation of another transporter of the SLC family, the excitatory amino acid transporter 2 (EAAT2), which normally rapidly retrieves glutamate to protect neurons from excitotoxicity, limiting glutamate re-uptake from the extracellular space, thus favoring seizure onset [84]. In addition, the upregulation of branched-chain amino acid transaminase 1 (BCAT1), which forms glutamate by transferring an α-amino group BCAAs to α-ketoglutarate, increases glutamate amount and release in IDH*^wt^* GBMs [85].

GBM cells highly express AMPA receptors (AMPAR, tetrameric assemblies of four GluR1–GluR4 subunits) but are often deficient (or low-expressing) in the Ca^2+^ impermeable GluR2 subunit [86]. Indeed, when the glutamatergic signal is blocked, survival, migration, and proliferation of glioma cells are impaired [87]. Glutamate exerts its tumorigenic effects via autocrine (activating glutamate receptors on GBM cells themselves) and paracrine (on adjacent astrocytes and neurons) stimuli and through the activation of AMPAR lacking GluR2, increasing Ca^2+^ influx and triggering growth-related MAPK and Akt pathways (Figure 2) [88,89]. AMPARs are Ca^2+^-permeable if they lack or contain the unedited GluR2, since in the brain, when this subunit is subjected to RNA editing (a post-transcriptional modification which converts a glutamine codon in the GluA2 gene in arginine codon in the mRNA), by the adenine deaminase RNA specific (ADAR2) enzyme, AMPARs are Ca^2+^-impermeable [90]. Thus, GluR2 RNA editing tunes glutamatergic neurotransmission and might play a key role in normal and pathologic brain, including glioma [91]. Interestingly, low ADAR2 protein in GBM cells promotes cell proliferation and migration and is associated with a patient’s shorter survival [92,93].

Overlapping epileptogenic and oncogenic mechanisms have been recently corroborated by the identification of functional synapses between GBM cells and neurons [94], forming tripartite glutamatergic synapses of cancer cells, and pre- and postsynaptic neurons [95]. These neuron–glioma networks are regulated by glutamate via AMPAR-dependent transmission and cancer-cell migration and proliferation, thus boosting GBM progression (Figure 1) [94,95].

Consequently, there is an emerging view based on glutamatergic signaling in neuron–GBM cell bidirectional interaction, which connects seizures and brain tumor progression and offers potential common therapeutic targets to treat both diseases.

### 4.5. Endogenous Cannabinoids

Endocannabinoid (eCB) system is composed of two major GPCR cannabinoid-specific receptors, namely CB1 and CB2, activated by eCBs (2-arachidoyl glycerol, 2-AG, and anandamide, AEA) and regulated by anabolic and catabolic enzymes. CB1 receptors are abundantly expressed in the CNS and on neurons, while CB2 receptors are principally present in the immune cells (e.g., leukocytes, microglia) [96] and other cell types, including cancer cells. Neuromodulatory signaling of eCBs is involved in a variety of brain physiological processes, and its alteration is relevant to psychiatric and neurological disorders, including epilepsy [97]. In animal models, eCB level is increased in mice with seizure-induced neurotoxicity (acute neuronal depolarization as by kainic acid, KA) [98,99], and deletion of CB1 in hippocampal glutamatergic excitatory neurons enhances seizure severity in KA-induced mice [100,101]. In epileptic patients, CB1 receptors and eCBs (i.e., AEA) are downregulated [102,103]. As previously highlighted, hyper-activation of glutamatergic transmission is a key pathogenic event for seizure generation; therefore, mechanisms regulating excitatory neurotransmission, such as the eCB system, might elucidate the pathophysiology of epilepsy and suggest novel therapeutic strategies.

As far as GBM, eCBs are still largely unexplored and contradictory: high 2-AG levels were detected in GBM [104,105], while AEA was found higher [106] or lower [107] in gliomas than in non-tumor tissues, and it could exert an in vitro and in vivo antitumor activity, as reported in GBM cell lines [104]. CB1 and CB2 receptors are variably present in human tumors; downregulation of CB1 levels in GBM, compared to a healthy brain, has been reported [108], as well as upregulation [105,108]. Concerning CB2, it is highly expressed in GBM [105], and antitumor effects of agonists have been reported in human GBM xenografts [109] and GBM stem cells [110].

Overall, evidence suggests a key role for eCB signaling in seizure onset and possibly as an add-on in resistant epilepsy [111], while its potential activity and mechanisms leading to GBM proliferation are largely unexplored and uncertain. 

## 5. Repurposing Antiepileptic Drugs for the Treatment of Glioblastoma: Pharmacologic Targets

The scenario so far described underlines critical point in GBM prognosis and management: (i) GBM has a fatal clinical course despite aggressive and multimodal treatments; (ii) epilepsy frequently develops in GBM patients; (iii) the two diseases show shared pathogenic processes.

Drug repurposing, identifying new therapeutic uses for already-available drugs, is a promising tool that speeds up drug discovery time. In GBM, several known compounds have been explored for new use as antitumor activity [112,113,114]. Therefore, repositioning AEDs can be a promising option able to improve patient survival and control both seizures and GBM growth and recurrence. 

LEV and VPA are the most studied drugs, possibly having a dual function as antiepileptic and antineoplastic, and are largely used in patients with GRE. Studies have also been carried out on other AEDs generally used as a second choice or add-on in case of therapeutic failure on the basis of the mechanism of action potentially impacting tumor growth (e.g., PER and CBD).

### 5.1. Antitumor Efficacy of AEDs Used in GRE: Preclinical and Clinical Perspectives

#### 5.1.1. Levetiracetam

LEV exerts its antiepileptic effects through binding to the synaptic vesicle glycoprotein 2A (SV2A), which regulates neurotransmitter vesicular dynamics in neurons, reducing Ca^2+^ release and acting as a negative allosteric modulator of GABA- and glycin-gated currents, thus supporting GABA release [115,116].

Antitumor efficacy of LEV, analyzed in GBM cell lines, has been attributed to the inhibitory action on MGMT, overcoming this GBM resistance mechanism and enhancing TMZ effects [117] by decreasing MGMT expression and activating apoptotic pathway [118]. Interestingly, LEV shows antitumor effects at concentrations in the serum therapeutic range for seizure prophylaxis [119]. In addition, LEV, combined with IFN-α, enhanced the anti-tumor activity of TMZ in MGMT-positive GBM cells [120].

In the clinical setting, the survival benefit of LEV has been evaluated in GBM patient treated with current SOC.

Retrospective analyses show that LEV improves patients’ PFS and OS [121,122,123]. However, a pooled analysis of four different clinical trials (NCT00943826, NCT00689221, NCT00884741, NCT00813943) on a large series of cases treated with LEV at the start of chemo-radio-therapy failed to find an association with patients’ survival [124]. This controversial association between LEV and clinical outcome was further investigated in an observational study on IDH*^wt^* GBMs, reporting a possible OS benefit when LEV is used during the whole standard SOC duration [125].

Overall, the observational retrospective studies contain information and selection bias, while prospective randomized controlled trials and studies including specific molecular profiles will help address the actual efficacy of LEV in GBM. Currently, an open-label single-arm phase 2 study, with external and historical control, enrolling 73 patients (NCT02815410), reported minimal survival benefits over the radio-chemo-therapy [126]. A study protocol for a double-blind randomized clinical trial focusing on the clinical benefits of LEV + TMZ in the treatment of GBM has been planned [127].

#### 5.1.2. Valproic Acid

VPA is a potent anticonvulsant acting through multiple mechanisms, such as interaction with GABA transaminase, succinate semialdehyde dehydrogenase, postsynaptic GABA and glutamate receptors, and ion channels [128]. Antitumor effects of VPA have been extensively explored in both the pre-clinical and clinical settings [129] being a histone deacetylase (HDAC) inhibitor (epigenetic drug) [130], changing not only in histone acetylation but also in DNA methylation in glioma cell lines [131].

Besides HDAC, other targets have been associated with VPA antitumor activity in GBM cells, such as the upregulation of brain-derived neurotrophic factor (BDNF) [132] and signaling pathways, such as ERK/Akt [133], Akt/mTOR [134], and Wnt [135]. In glioma cells, VPA promotes apoptosis [133,134] and autophagy [134,136] and impairs cell proliferation and invasiveness [135,137]. However, the effective dose (millimolar) of VPA is far above that used for the treatment of epilepsy [138]. At a concentration of up to 100 µM, VPA failed to inhibit glioma cell growth and metastasis in vitro [139]. Beyond antitumor effects as a single agent, VPA sensitizes GBM cells to several anticancer drugs, such as nitrosoureas [140], TMZ [141,142,143], gefitinib [144], etoposide [145], and radiation therapy [143,146].

Therefore, based on in vitro and in vivo preclinical results, VPA may manage both epilepsy and glioma as a potential adjuvant drug to enhance patients’ response to SOC. 

Several studies show that VPA increases the median survival of patients affected by GBM [147,148,149]. Retrospective studies suggest that adding VPA to TMZ-based chemoradiotherapy in newly diagnosed GBMs slightly prolongs survival at the expense of increased thrombocytopenia and leukopenia [148,149,150]. In addition, VPA used during radiotherapy decreases side effects and prolongs OS and PFS [151].

A recent update of an open-label phase 2 study (NCI-06-C-0112) on 37 patients confirms previous data reporting that the addition of VPA improves their PFS and OS [152]. A meta-analysis confirmed the prolonged survival with VPA combined treatment [153]; conversely, analysis of pooled randomized trials enrolling 1869 patients did not associate with extended PFS or OS [124]. 

As a whole, preclinical evidence supports VPA antitumor and TMZ-sensitizing efficacy, while in the clinical setting, further investigation is needed to justify VPA as a potential adjuvant in current GBM therapy.

#### 5.1.3. Perampanel

PER is a selective non-competitive AMPA receptor antagonist. The shared mechanism of AMPA-activation connecting seizure activity and GBM growth support the rationale for AMPA-receptor blocker evaluation of antitumor activity, first investigated with talampanel, a PER analog, which is still not on the market due to its poor pharmacokinetics and short half-life [154], without reaching concordant significant results [155,156,157].

The antiproliferative mechanism of PER is not yet clear and preliminary data are obtained in a wide range of concentrations and different established glioma cell lines. In a study comparing antitumor effects in GBM cell lines of various AEDs, including LEV and VPA, only PER showed inhibitory effects on cell proliferation, migration, and invasion, without induction of apoptosis and reduction of extracellular glutamate levels [139,158]. Whereas, in other works, PER antineoplastic activity is mediated by apoptosis, possibly due to increased GluR2/3 expression synergizing with TMZ [159,160].

In in vivo experiments in C6 rat glioma xenografts, PER did not affect either tumor growth or animal survival, while it blocked epileptiform discharges in organotypic glioma slices and reduced glucose uptake in C6 glioma cells in vitro [161]. Similarly, in another orthotopic rat model of glioma (F98), which promotes an epileptiform phenotype, the therapeutic efficacy of PER, as an adjuvant to standard radiochemotherapy, failed to impact tumor progression and animal survival, while tumor-associated epilepsy was abolished, maintaining the glutamatergic network activity on healthy peritumoral tissue of treated animals [162].

Interestingly, in the scenario of neuro-glioma synapses which produce postsynaptic currents mediated by AMPA receptors, the 14-day treatment of GBM xenografted mice with PER exerts significant antiproliferative activity on GBM cells [95] and in mice bearing pediatric glioma [94].

At the clinical level, only one retrospective study evaluated PER impact on seizures and tumor progression in 12 GBM patients with refractory epilepsy, detecting by MRI a reduction of tumor volume [163]. Currently, while in the management of GRE, PER in add-on could be a valid therapeutic option, and a study is ongoing to confirm its safety and efficacy (NCT04650204); trials evaluating PER efficacy on GBM progression and survival are lacking.

However, the connection between increased neuronal activity and glioma progression mediated by glutamatergic synapses warrants further investigation in which AMPA receptor inhibition by PER may be exploited both at the preclinical and clinical levels.

#### 5.1.4. Cannabidiol 

Cannabidiol (CBD) is one of the major molecules found in cannabis and hemp, and pharmaceutical-grade CBD has FDA and ENMA approval for seizures in Dravet syndrome and Lennox–Gastaut syndrome, as well as for tuberous complex [164].

Although the mechanism of action is not yet fully understood, CBD has numerous targets; in particular, its anticonvulsant activity is due to the interaction with three receptors—the transient receptor potential vanilloid-1 (TRPV1), the orphan G protein-coupled receptor-55 (GPR55) and the equilibrative nucleoside transporter 1 (ENT-1), implicated in the regulation of neuronal excitability [165].

The in vitro and in vivo anti-glioma activity of different CB1/CB2 agonists (cannabinoids) and endocannabinoids has been described (for a review, see [97]). 

Phytocannabinoids, namely CBD, in preclinical studies, demonstrated antiproliferative and pro-apoptotic effects and inhibition of the migration of GBM [166], acting as a sensitizer to chemotherapeutic agents [167]. In human primary GBM stem-like, CBD exerts cytotoxic activity likely through modulation of a key transcription factor in GBM, the nuclear factor kappa B (NF-κB), by promoting DNA binding and preventing posttranslational modification of the NF-κB subunit RELA/p65 [168]. 

In a retrospective trial, CBD seems to demonstrate an increase in the survival of patients with GBM [169].

Results from a phase 1b randomized trial of cannabinoid oromucosal spray (nabiximols, containing delta-9-tetrahydrocannabinol-THC, CBD, and additional cannabinoid and non-cannabinoid components) with TMZ in patients with recurrent GBM showed satisfactory safety and tolerability, no drug–drug interaction, and survival at 1 year of 83% for nabiximols and 44% for placebo arm [170].

Currently, there is an ongoing phase 1b trial (NCT03529448) assessing the safety and antitumor activity of the THC + CBD combination with standard therapy in newly-diagnosed GBM.

Further studies will show whether compounds that exert promising effects on tumor cells, in addition to the psychoactive THC, such as CBD and inhibitors of endocannabinoid degradation, could be potential combination partners for established chemotherapeutic agents in GBM treatment.

### 5.2. Potential Repositioning of Other AEDs Used in GRE

Few in vitro investigations report on the potential antitumor effects of other approved AEDs used in GRE.

Among them, LCM has been shown to be ineffective [171] or to exert cytotoxicity and anti-migratory effects in the micromolar range [172] in GBM cells lines, likely via HDAC inhibition, as observed in a breast cancer model [173]. Curiously, the inactive enantiomer of this AED, S-lacosamide (S-LCM), is able to decrease GBM cell proliferation and the growth of orthotopic tumors in a murine GBM model [174].

BRV, with a 15- to 30-fold higher affinity for SV2A than LEV, shares the above-described antitumor effects with LCM [172].

LMG fails to reach significant cytotoxicity at therapeutic concentrations in vitro [138]; however, P3K/AKT signaling might represent its additional target, also blocking voltage-dependent calcium and sodium channels, to exploit antitumor effects as observed in breast cancer models [175].

PGB, a GABA analog, exhibits anti-neuroinflammatory effects, preventing substance P (SP)-mediated IL-6 and IL-8 production in the U373 GBM cell line through the inhibition of p38 MAPK and NF-κB signaling molecules [176].

Similarly, a slight cytotoxic activity in GBM cells has been described for TPM [138].

Stiripentol, an agonist of GABA_A_R, approved for the treatment of seizures associated with Dravet syndrome [177], showed selective cytotoxic and anti-migratory activity in GBM cells and additive or synergistic effects with TMZ [178]. Interestingly, stiripentol decreases GBM invasion and growth in xenografted mice [179], likely via lactate dehydrogenase (LDH) block, an enzyme involved in both neuron hyperpolarization [180] and highly glycolytic metabolism of GBM cells, which catalyzes lactate production and correlates with high proliferation and invasion [181].

Comprehensive antitumor mechanisms of the above AEDs remain unclear, as well as their efficacy in vivo; however, several mechanisms might represent a novel therapeutic approach for targeting GBM (Table 1).

## 6. Conclusions

**Figure 3:**
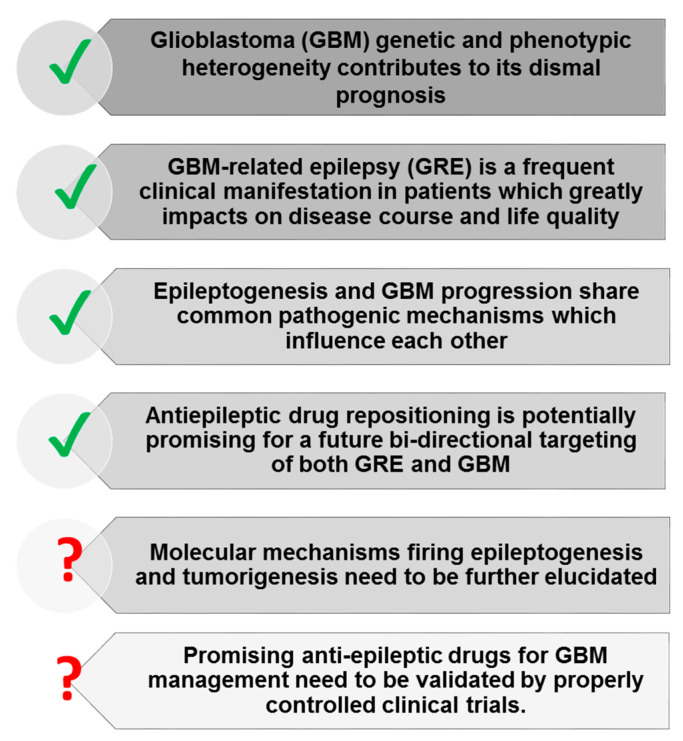
Antiepileptic drugs in human glioblastoma—a quick overview of current knowledge and future perspectives.

GBM is a lethal, highly proliferating, and invasive cancer that tightly interconnects with brain tissue, coopting astrocytes, macrophages/microglia, and endothelial cells in the tumor microenvironment, regulated by both brain activity and neuron–glioma synaptic interaction (Figure 3). Communication may occur directly between neuron and glioma cells or by regulation of other cell types within the microenvironment through neurotransmitter signaling. Neuronal mechanisms can trigger GBM cell growth and invasion and sustain intratumoral cellular heterogeneity. Concomitantly, cellular and molecular alterations in GBM cells and peritumoral tissues may generate epileptic symptoms in patients with GBM via shared signals mediated by neurotransmitters (glutamate, GABA).

Further studies are issued to clarify the anti-tumor efficacy of AEDs based on specific biomarkers of GBM so that two diseases, GRE and GBM, could be targeted with significant clinical benefit simultaneously (Figure 3). The emerging research field of cancer neuroscience will help identify specific and mutual pathophysiology and vulnerabilities of GBM progression and seizure onset, potentially promising for future bidirectional therapeutic targeting of common potential drivers, such as glutamatergic signaling, as studies with different AEDs, although presently not conclusive, suggest.

## Figures and Tables

**Table 1:** Suggested targets and antitumor efficacy of antiepileptic drugs in GBM.

Drug	Antiepileptic Targets	Antitumor Targets	Preclinical Studies	Clinical Studies	#Clinical Trials *
Brivaracetam	Na^+^ channels SV2A GABA	MGMT	[172]	[51]	-
Cannabidiol/cannabinoids	TRPV1 GPR55 ENT-1	NF-κb	[166,167,168]	[169,170]	NCT03529448 ^NR^ NCT03607643 ^NR^ NCT03529448 ^NR^ NCT0181260 ^C^ NCT01812616 ^C^
Carbamazepine	Na^+^ channels	u.k.	[158]	-	-
Lamotrigine	Na^+^ channels Ca^2+^ channels	PTEN PI3K/Akt	[138]	-	-
Lacosamide	Na^+^ channels	HDAC CRMP2	[172,174]	-	-
Levetiracetam	SV2A Ca^2+^ channels GABA	MGMT HDAC	[117,118,119,120,171]	[121,122,123,125,126,182]	NCT03048084 ^R^ NCT00629889 ^C^ NCT02815410
Perampanel	AMPAR	AMPA/glutamate	[139,160,159]	[163]	NCT04650204 ^R^
Pregabalin	α2δ subunit Ca^2+^ channels	p38 MAPK NF-κB	[176]	-	NCT00629889 ^C^
Stiripentol	GABA_A_R	LDH	[178,179]	-	-
Topiramate	Na^+^ channels GABA_A_R AMPA/kainateR Ca^2+^ channels	u.k.	[138]	-	-
Valproic Acid	GABA Na^+^ channels NMDAR Ca^2+^ channels	HDAC BDNF ERK/Akt Akt/mTOR Wnt	[133,134,135,136,137,138,140,141,142,143,144,145,146,171]	[147,148,149,150,151,182,183]	NCT01817751 ^NR^ NCT03243461 ^R^ NCT03048084 ^R^ NCT00879437 ^C^ NCT00302159 ^C^ NCT02648633 ^C^

* Clinicaltrials.gov (accessed on 10 January 2023); ^NR^, not recruiting; ^R^, recruiting; ^C^, completed. ***Abbreviations:*** u.k. = unknown; SV2A = synaptic vesicle glycoprotein 2A; GABA = γ-Aminobutyric acid; TRPV1 = transient receptor potential vanilloid-1; GPR55 = orphan G protein-coupled receptor-55; ENT-1 = equilibrative nucleoside transporter 1; AMPA = α-amino-3-hydroxy-5-methyl-4-isoxazolepropionic acid; α2δ = alpha2-delta protein, an subunit of voltage-gated Ca^2+^ channels; NMDA = N-methyl-D-aspartate; MGMT = O(6)-methylguanine-DNA methyltransferase; NF-κb = nuclear factor kappa B; HDAC = histone deacetylase; PTEN = phosphatase and TENsin homolog; PI3K = phosphatidylinositol-3 kinase; AKT = Protein kinase B; CRMP2 = collapsin response mediator protein 2; p38 MAPK = 38-kDa mitogen-activated protein; LDH = lactate dehydrogenase; BDNF = brain-derived neurotrophic factor); ERK = extracellular signal-regulated kinase; mTOR = Mammalian target of rapamycin; Wnt = Wingless/Integrated pathway.

## Data Availability

Not applicable.
